# Applications of Hydrophilic Interaction Chromatography in Pharmaceutical Impurity Profiling: A Comprehensive Review of Two Decades

**DOI:** 10.3390/molecules30173567

**Published:** 2025-08-31

**Authors:** Marianna Ntorkou, Constantinos K. Zacharis

**Affiliations:** Laboratory of Pharmaceutical Analysis, Department of Pharmacy, Aristotle University of Thessaloniki, 54124 Thessaloniki, Greece; marianna.ntorkou98@gmail.com

**Keywords:** impurity profiling, separation, detection, HPLC, active pharmaceutical ingredient, review

## Abstract

Hydrophilic Interaction Liquid Chromatography (HILIC) has emerged as a powerful and versatile analytical technique for the separation and quantification of polar and ionizable compounds, particularly in the field of pharmaceutical impurity profiling. Over the past two decades, HILIC has gained increasing attention due to its compatibility with mass spectrometry, enhanced retention of hydrophilic impurities, and ability to resolve structurally similar degradation products and process-related impurities that are often inadequately retained by reversed-phase chromatography. This comprehensive review presents a critical overview of HILIC-based methodologies applied to impurity profiling in pharmaceutical analysis from early 2005 to the present. Emphasis is placed on the application of HILIC to both small-molecule drugs and large biomolecules. Additionally, the review categorizes analytical configurations into four main groups based on their operational principles and use cases, offering insights into method selection and performance characteristics. This article serves as a valuable resource for researchers and regulatory scientists seeking to apply HILIC in modern impurity profiling and quality control of pharmaceutical products.

## 1. Introduction

The primary objective of the pharmaceutical industry is to protect public health by delivering safe and effective medications. To achieve this, it is essential to maintain high-quality standards throughout the entire lifecycle of pharmaceutical materials, ensuring that the final products meet their intended purpose [1,2].

Regulatory frameworks, such as those established by the United States Pharmacopeia (USP) and the International Council for Harmonization (ICH), classify drug impurities into categories including residual solvents, as well as organic and inorganic impurities [1]. Within the pharmaceutical industry, various terms are used to describe organic impurities, such as intermediates, starting materials, final intermediates, byproducts, transformation products, related substances, degradation products, and interaction products. Recognizing the potential risks these impurities pose to human health, regulatory authorities worldwide have implemented stringent guidelines to monitor and control their levels in active pharmaceutical ingredients (APIs) and drug products [1,3,4,5,6]. A recent review by C.F. Cordeiro et al. [7] provides an updated overview of impurities and the associated regulatory requirements for their control in pharmaceuticals. The detection and quantification of these impurities, often present at trace levels, remain a significant analytical challenge, requiring the development of highly selective and sensitive analytical techniques and methods.

Separation techniques are essential for the identification and quantification of impurities in pharmaceutical products. Among others, high-performance liquid chromatography (HPLC) is the main analytical technique used for the determination of thermally unstable and non-volatile impurities [8]. Recent advancements in chromatographic column technology, hardware improvements, new types of detectors, and the integration with various orthogonal techniques have significantly enhanced the capabilities of HPLC. These developments have broadened its application, making it a powerful tool for detecting impurities and degradation products with diverse physicochemical properties [9,10]. Looking at the stationary phases, various types have been developed and commercialized including hydrophobic (C18, C8, phenyl, perfluorophenyl), ion exchange (cation exchange, anion exchange), hydrophilic interaction chromatography (HILIC), etc. [10]. In addition to single-mode stationary phases—where a single mechanism is typically process—mixed-mode phases (e.g., hydrophilic interaction/cation exchange) have also been developed, offering high separation selectivity, remarkable loading capacity, and excellent separation efficiency [11].

HILIC has emerged as a powerful technique for the separation of polar and hydrophilic compounds that are often poorly retained in traditional reversed-phase high-performance liquid chromatography (RP-HPLC). This mode of chromatography has gained widespread attention due to its high separation efficiency, enhanced sensitivity in mass spectrometry applications, and compatibility with a wide range of biological and pharmaceutical samples. As the demand for improved analysis of polar compounds continues to grow, HILIC has become an essential tool in fields such as metabolomics, proteomics, and pharmaceutical analysis [12,13,14]. A search of literature using Scopus shows a significant rise in publications on HILIC between 2014 and 2022, with over 550 articles published annually. Most of these publications focus on the diverse applications of HILIC, highlighting the value of this distinctive chromatographic technique.

To the best of our knowledge, several review articles have been published on the application of HILIC to the analysis of pharmaceuticals [15,16,17], mainly focusing on the determination of APIs in pharmaceutical products or biological matrices. Moreover, three review articles have recently been published focusing on the use of HILIC for the analysis of oligonucleotides and their related impurities [18,19,20]. In contrast, the present comprehensive review emphasizes HILIC-based analytical applications for the quantification of impurities in both small- and large-molecular-weight drugs over the past two decades. The article is structured into four main sections, each highlighting different HILIC-based analytical configurations employed for impurity profiling.

## 2. HILIC

### 2.1. General Considerations

HILIC was pioneered by A. J. Alpert to address the challenge of poor retention of polar compounds in RP-HPLC [21]. He described HILIC as a chromatographic technique where a hydrophilic column is eluted using a predominantly organic (hydrophobic) mobile phase, resulting in greater retention for more hydrophilic solutes. He proposed the term “hydrophilic-interaction chromatography” to distinguish this method as a variation of normal-phase chromatography.

**Figure 1:**
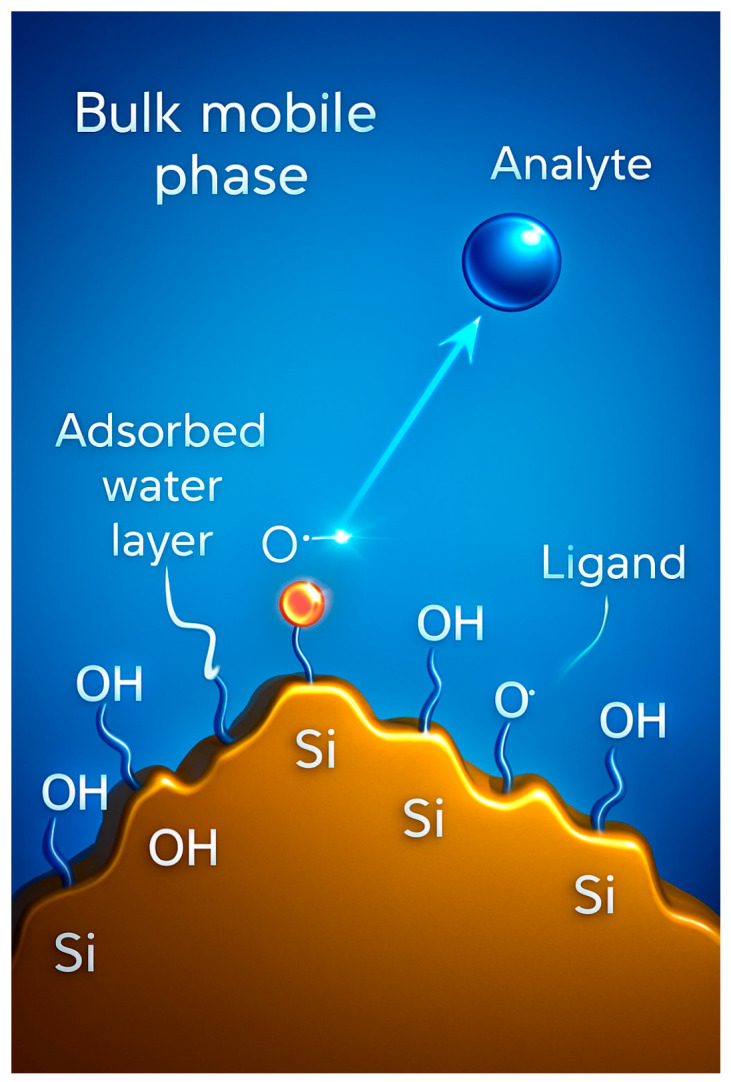
Illustration of the HILIC (hydrophilic interaction chromatography) stationary phase, showing the adsorbed water layer, attached ligands, and the surrounding bulk mobile phase.

Many efforts have been made to understand HILIC mechanisms [22]. It was proposed that there is a partition of a water-enriched layer partially immobilized on the stationary phase and the mobile phase, which is mainly a polar organic solvent [23]. Figure 1 schematically represents the interactions in HILIC.

Other mechanisms, including surface adsorption and electrostatic interactions, also significantly contribute to retention. Their importance varies based on factors such as the content of organic solvent. More recently, Guo’s research group reported advancements in understanding the HILIC mechanism, with a focus on key aspects such as the water layer adsorbed onto the stationary phase, the selectivity of various stationary phases, the kinetic performance, and the role of electrostatic interactions between ionized analytes and charged stationary phases [24,25,26,27].

A variety of polar stationary phases are available for HILIC, supporting its continued use in pharmaceutical analysis over the past few decades. Choosing a stationary phase in HILIC can be more complex than in RPLC, where partition is the dominant mechanism, and retention on phases such as C18 and C8 can be accurately predicted based on compound polarity. Manufacturers have created phases with varying chemistries to support specific mechanisms, including materials that can function as either RP or HILIC phases (depending on the mobile phase) as well as columns containing ionic groups. Over the years, classical bare silica, silica gels, and polymer-based materials modified with various polar functional groups have been developed. Among others, diol-, cyano-, amino- amide-, zwitterionic sulfobetaine-bonded phases, cationic exchangers, mixed-mode RP/anionic exchangers, and non-conventional materials (i.e., ionic liquid, carbon dots) have been developed. The characteristics and applications of various HILIC stationary phases have been comprehensively examined in the literature [23,28,29,30].

Looking at the mobile phases, mixtures of an aprotic water-miscible solvent (i.e., acetonitrile (ACN), tetrahydrofuran) with a small amount of water containing ionic additives (ammonium acetate (NH_4_Ac) or formate) are the most preferable mobile phases for HILIC separations [23]. Parameters such as the type of organic solvent, pH, and salt concentration strongly affect analyte retention [29]. Acetonitrile (ACN) is the most used organic solvent, while the aqueous component typically contains salts and acids to regulate pH and ionic strength. These additives should be volatile to prevent issues at the interface with MS-based detectors [26]. The key principles, effects, and current trends related to mobile phase optimization in HILIC have been thoroughly reviewed [31].

### 2.2. Structure-Guided Stationary Phase Selection

Understanding the relationship between the analyte structure and *stationary phase* is essential for rational *HILIC* method development. While empirical trial and error remains common practice, general structural rules can provide valuable guidance in selecting suitable stationary phases. The following guidelines summarize how key structural features influence HILIC retention and how stationary phase chemistry can be selected accordingly [14,16,32].

(i) Neutral polar groups (–OH, –NH_2_, –CONH_2_): Compounds containing neutral polar groups interact strongly with the water-enriched layer on HILIC stationary phases. For example, nucleosides and “sugar-like” drugs (i.e., macrolide drugs) often exhibit robust retention on bare silica or zwitterionic phases due to hydrogen bonding and partitioning into the aqueous layer.
(ii) Acidic groups (–COOH, –SO_3_H): Carboxylic acids and sulfonic acids exhibit retention through both hydrophilic partitioning and electrostatic interactions. Their behavior is highly pH-dependent: under partially ionized conditions, acidic analytes benefit from zwitterionic or amino-type phases that provide complementary ionic interactions.
(iii) Basic groups (–NH_2_, heteroaromatic nitrogens): Basic analytes (e.g., many antibiotics, nucleobases) are prone to strong electrostatic interactions with residual silanols, leading to poor peak shape if not properly controlled. Zwitterionic or amide-type HILIC phases can minimize this issue by balancing ionic and hydrogen-bonding interactions, improving reproducibility.
(iv) Zwitterionic or amphoteric compounds: Molecules with both acidic and basic groups (e.g., amino acids, peptides) benefit from stationary phases that contain zwitterionic ligands. These phases stabilize both charge states and offer consistent retention while minimizing peak tailing.

By aligning stationary phase chemistry with the dominant functional groups of an analyte, HILIC method development can become more predictable and less reliant on empirical screening. This structure-guided strategy helps ensure optimal selectivity, retention, and peak shape in the analysis of highly polar compounds.

## 3. Applications

### 3.1. HPLC-UV/FLD

In HILIC, UV detection is particularly suitable for small polar molecules with UV-active functional groups. While UV detectors offer good linearity and are easy to couple with chromatographic systems, their effectiveness is limited for analytes lacking chromophores. Moreover, the composition of the mobile phase, especially high organic content in HILIC, must be carefully considered to avoid baseline noise or drift. Despite these limitations, UV detection remains a preferred choice for routine analysis in pharmaceutical and bioanalytical applications. On the other hand, fluorescence detectors offer high sensitivity and selectivity, making them particularly useful for trace analysis in complex pharmaceutical matrices. An overview of HILIC-UV/FLD applications is summarized in Table 1.

Locatelli et al. developed a HILIC-UV method for the simultaneous determination of prulifloxacin, its active metabolite ulifloxacin, and four related synthetic process impurities in a tablet formulation [33]. The separation was carried out on a Luna HILIC column under isocratic elution mode with NH_4_Ac buffer and ACN. This system allows increased retention of polar compounds in a highly organic mobile phase. Compared to other previously reported methods for the determination of prulifloxacin, this method required minimal sample preparation and proved to be accurate and selective for the simultaneous determination of the six analytes.

A. Szterk and colleagues developed a HILIC-UV method for quantifying succinylcholine and its impurities in both API and finished medicinal products [34]. Detection was performed at 214 nm, and isocratic separation was achieved using a mobile phase of 30% phosphate buffer (pH 4.0, 0.05 M) in ACN. The method demonstrated high resolution (R_s_ > 3) between succinylcholine and its primary impurity, succinylmonocholine, with narrow peak widths (≤0.7 min). LODs were low, measured at 2.4, 6.0, and 11.5 μg/mL for succinic acid, succinylmonocholine, and succinylcholine, respectively. Excellent linearity was observed across a broad concentration range (7.3–670 μg/mL; R^2^ = 0.999). Recovery at three fortification levels ranged from 95.7% to 98.9%. Precision (intra- and inter-day) ranged from 1.0% to 5.9% CV, while accuracy ranged from 1.3% to 6.3%. Furthermore, previously unidentified impurities in the API were detected using ultra-high-performance liquid chromatography coupled with quadrupole time-of-flight mass spectrometry (UHPLC-QTOF-MS).

The research group of A. Kumar from Micro Labs Ltd.’s API R&D center established a sensitive and selective HILIC-UV protocol for the separation and quantification of five potential genotoxic impurities in dalfampridine dosage forms [35]. The method achieved lower LODs for all impurities, compared to other reported approaches [36,37]. After thorough investigation, a Zorbax Silica (250 × 4.6 mm, 5 μm) column was finally chosen, since it provided good retention of the analytes, better peak shape and separation than C_8_, C_18_, ZIC-HILIC and cyano columns, with peak resolution values ranging from 4.34 to 12.77. Moreover, the aforementioned column resulted in improved recovery values (88.1–107.7%) for all analytes compared to the method using an ion-pairing reagent as reported in the USP. The ion-pairing approach led to saturation of the active ionic sites on the HPLC column by dalfampridine, likely due to its significantly higher concentration (20,000 μg/mL) relative to the potential genotoxic impurities. The developed HILIC method proved to be suitable and versatile for routine analysis of production samples and stability checking of dalfampridine dosage forms.

**Table 1:** HPLC-UV/FLD applications for the determination of drug impurities.

Analyte	Analytical Column	Mobile Phase	Flow Rate (mL/min)	UV/FLD Wavelength(s) (nm)	LODs/LOQs	Recovery (%)	Reference
Prulifloxacin, ulifloxacin and four process impurities	Diol-bonded silica (250 × 4.6 mm, 5 μm)	NH_4_Ac buffer (5 mM, pH = 5.8) and ACN	1	218–283 (UV)	0.15/0.25 μg/mL (3/5 μg/mL for ulifloxacin)	NA	[33]
Amino acids and amino acid-like molecules	Diol-bonded core–shell silica (100 × 4.6 mm, 2.5 μm)	Potassium phosphate buffer (12.5 mM, pH = 2.8) and ACN	1.4	200 (UV)	0.02–8.0/0.05–20.0 μg/mL	97.3–110.6	[38]
Polar API and three impurities	Diol-bonded silica (250 × 4.6 mm, 5 μm) C_18_ (100 × 4.6 mm, 3.5 μm)	0.09% phosphoric acid and ACN Ammonium chloride (10 mM) and ACN	1.5 1.0	215 (UV)	NA/0.05% (for impurities)	99.2–100.6	[39]
Bilastine and two impurities	Diol-bonded silica (100 × 4.6 mm, 5 μm)	NH_4_Ac buffer (50 Mm, pH = 5.3)	1.0	275 (UV)	0.16/0.5 μg/mL (for impurity 1) 0.08/0.25 μg/mL (for impurity 2)	98.7–101.8	[40]
Acetylsalicylic acid, amlodipine, one impurity and atenolol	Diol-bonded silica (100 × 4.6 mm, 2.6 μm, 100 Å)	NH_4_Ac buffer (75 mM, pH = 5.3) and ACN	1.0	254 (UV)	NA/NA	NA	[41]
Succinylcholine and impurities	HILIC–silica (250 × 4.6 mm, 5 μm)	30% phosphate buffer (pH = 4.0; 0.05 M) in ACN	1.0	214 (UV)	2.4–11.5/7.3–24.9 μg/mL	95.7–98.9	[34]
Sodium cromoglicate	HILIC–silica (250 × 4.6 mm, 5 μm)	NH_4_Ac buffer (30 mM, pH = 3.0) and ACN	2.0	326 (UV)	25/100 ng/mL	100.4	[42]
Dalfampridine and five process impurities	Bare silica (250 × 4.6 mm, 5 μm)	Ammonium formate (10 mM, pH = 5.0) and MeOH:ACN (10:90 *v*/*v*)	1.0	280 (UV)	7.19–7.64/21.57–22.93 μg/mL	88.1–107.7	[35]
Linagliptin and its impurity 3-aminopyridine	EXTRASIL Silica (150 × 4.0 mm, 3 μm)	NH_4_Ac buffer (10 mM, pH = 6.0) and ACN	1.0	298 (UV)	7.5/25 μg/mL (with respect to linagliptin)	97.3–101.3	[43]
Metformin hydrochloride and its related impurities, cyanoguanidine and melamine	HILIC–silica (250 × 4.6 mm, 5 μm)	Sodium phosphate buffer (25 mM, pH = 3.0) and ACN	2.0	218 (UV)	5.0–100/25–350 ng/mL	98.1–101.0	[44]
2-aminoisobutyric acid	HILIC–silica (250 × 4.6 mm, 5 μm)	Potassium acetate buffer (25 mM, pH = 5.5) and ACN	0.8	345 (excitation)/ 450 (emission) (FLD)	4.5/15 ng/mL	99.9	[45]
Moxonidine and four impurities	RX–silica (250 × 4.6 mm, 5 μm)	Ammonium formate buffer (40 mM, pH = 2.8) and ACN	1.0	255 (UV)	0.012/0.04 μg/mL (for impurities A,B) 0.024/0.08 μg/mL (for impurities C,D)	93.7–114.1	[46]
Salicylic acid, acetyl salicylic acid and Ascorbic acid	RX–silica column (250 × 4.6 mm, 5 μm)	NH_4_Ac buffer (22 mM, pH = 4.4) and ACN	N/A	285 (UV)	0.03 μg/mL (for salicylic acid)/0.09–2.5 μg/mL	93.9–105.8	[41]
Iodixanol and three impurities	Core–shell silica (150 × 4.6 mm, 2.6 μm)	Formic acid solution (1 mM, pH = 3.2) and ACN	0.8	243 (UV)	0.024–0.19/0.061–0.48 μg/mL	97.5–103	[47]
Tofacitinib citrate and related substances	Zwitterionic HILIC (250 × 4.6 mm, 5 μm)	Potassium phosphate buffer (10 mM, pH = 7.0) and ACN	0.5	210 (UV)	0.03/0.05 and 0.06% *w*/*w*	86.0–100.0	[48]
Sulfaquinoxaline sodium and related compound A	Zwitterionic HILIC (250 × 4.6 mm, 5 μm)	NH_4_Ac buffer (200 mM, pH = 5.7) and ACN	0.5	263 (UV)	0.04/0.13 μg/mL (for sulfaquinoxaline sodium)	98.9–100.9	[49]
Iohexol, one isomer and three impurities	Zwitterionic HILIC (100 × 4.6 mm, 5 μm)	NH_4_Ac buffer (72 mM, 6.5) and ACN	1.0	254 (UV)	0.08/0.25 μg/mL	94.2–110.2	[50]
*N*-hydroxysuccinimide and *N*-hydroxysulfosuccinimide	Ζwitterionic silica-based HILIC (150 × 3 mm, 3 μm)	NH_4_Ac buffer (10 mM, pH = 7.5) and ACN	0.4	220 and 260 (UV)	1/3 mg/L (for *N*-hydroxysuccinimide) and 0.5/1.5 mg/L (for *N*-hydroxysulfosuccinimide)	NA	[51]
Sildenafil citrate and its impurity imidazole	Zwitterionic sulfobetaine-bonded silica (150 × 4.6 mm, 5 μm, 200 Å)	NH_4_Ac buffer (10 mM, pH = 5.5) and ACN	0.85	210 (UV)	0.025/0.125 μg/mL (for imidazole)	NA	[52]
*N,N’*-ethylenebis-l-cysteine diethyl ester (Bicisate) and impurities	Mixed-mode HILIC (150 × 4.6 mm, 5 μm, 100 Å)	TFA solution (7.5 mM) and ACN	1.0	215 (UV)	NA/NA	85.0–109.0	[53]
l-ascorbic acid 2-phosphate magnesium	Mixed-mode HILIC (150 × 3.2 mm, 5 μm)	Potassium phosphate buffer (15 mM, pH = 2.5) and ACN	0.4	240 (UV)	0.017/0.052%	97.1–101.2	[54]
Capreomycin sulfate and four impurities	Mixed-mode HILIC (250 × 4.6 mm, 5 μm)	Ammonium trifluoroacetate buffer (20 mM, pH 5.0) and ACN	1.0	260 (UV)	NA/NA	NA	[55]
2-chloromalonaldehyde	Amide HILIC (150 × 2.1 mm, 1.7 μm)	NH_4_Ac buffer (200 mM) and ACN	0.5	273 (UV)	0.004/0.01 mg/mL	79.1–106.6	[56]
Amlodipine besylate and three impurities	Aminopropyl-bonded silica (250 × 4.6 mm, 5 μm)	NH_4_Ac buffer (50 mM, pH = 4.0) and ACN	1.0	230 (UV)	0.010/0.025 μg/mL	96.4–101.2	[57]
Amitriptyline and six impurities	Aminopropyl-bonded silica Amide-bonded silica Bare silica Diol-bonded silica (250 × 4.6 mm, 5 μm)	NH_4_Ac buffer (various pH values) and ACN	1.0	254 (UV)	NA/NA	NA	[58]
Amlodipine besilate and two impurities bisoprolol fumarate and four impurities	Aminopropyl-bonded silica (250 × 4.6 mm, 5 μm) Bare silica (100 × 4.6 mm, 5 μm) Diol-bonded silica (100 × 4.6 mm, 5 μm, 200 Å)	NH_4_Ac buffer (20 mM, pH = 4.0) and ACN	1.0	230 (UV)	NA/NA	NA	[59]
Amitriptyline and four impurities	Aminopropyl-bonded silica (100 × 4.6 mm, 5 μm)	NH_4_Ac buffer (60 mM, pH = 4.5) and ACN	1.0	254 (UV)	0.025–0.50/0.05–1 μg/mL	96.4–102.1	[60]
Eleven nicotinamide metabolites	Pentyl-bromide (3PBr) (150 × 3 mm, 3 μm)	Ammonium formate buffer (20 mM, pH = 6.4) and MeOH	0.4	260 (UV)	NA/NA	NA	[61]
Four cyclic synthetic pharmaceutical peptides	Silica-based (150 × 2.1 mm, 1.7 μm) Acidic (tetrazole), basic (pyridyl), zwitterionic (phosphorylcholine) (250 × 4.6 mm, 3 µm)	NH_4_Ac buffer (20 mM, pH = 4.7) and ACN	1.0	220 and 254 (UV)	NA/NA	NA	[62]
Olopatadine hydrochloride, one isomer and benzalkonium chloride	Cyano-bonded silica (100 × 4.6 mm, 5 μm)	NH_4_Ac buffer (5 mM, pH = 4.5) and ACN	1.0	257 (UV)	0.1/0.375 μg/mL (for isomer) 6/20 μg/mL (for benzalkonium chloride)	83.2–101.5	[63]
Acarbose and seven impurities	Pentafluorophenyl (PFP)-bonded core–shell silica (100 × 4.6 mm, 2.6 μm)	Ammonium formate buffer	1.0	210 (UV)	NA/NA	NA	[64]
Oxaliplatin enantiomers	Cellulose-based silica (100 × 4.6 mm, 3 μm)	Water and ACN	1.0	210 (UV)	0.07/0.21 μg/mL	NA	[65]

The genotoxic impurity profile has also attracted the attention of B. Al-Sabti and J. Harbali. They developed an analytical method for the quantitative determination of a potential genotoxic impurity 3-aminopyridine in the linagliptin API using HILIC-UV [43]. 3-aminopyridine is a reactive reagent in the synthesis of linagliptin. The chromatographic conditions included a mobile phase consisting of 10 mM NH_4_Ac buffer and ACN at a ratio of 10:90 *v/v* adjusted at pH 6.0, at a flow rate of 1 mL/min and a TRACER EXTRASIL Silica (150 × 4.0 mm, 3 μm) (Teknokroma S.A., Barcelona, Spain) column thermostated at 25 °C. The method was applied to monitor the 3-aminopyridine impurity in linagliptin during its synthesis, as well as in the testing of linagliptin raw materials as part of quality control prior to their use in pharmaceutical manufacturing.

Another published study deals with the development and validation of a HILIC-UV method for the separation of some underivatized hydrophilic amino acids and amino acid-like molecules, namely aspartic acid, carnitine, creatinine, arginine and the tripeptide glutathione, in complex alimetary supplement formulations [38]. Due to the oxidative susceptibility of glutathione and its low concentration in pharmaceutical formulations, a reference RP-HPLC method was also introduced for the selective determination of the active thiol, employing 1,4-naphthoquinone as a pre-column derivatization reagent. Separations were performed on a Phenomenex Kinetex core–shell 2.6 μm HILIC (100 × 4.6 mm i.d.) (Phenomenex Technologies, Torrance, CA, USA)column with a mobile phase consisting of ACN/potassium phosphate monobasic in gradient elution mode, while in the analysis of GSH by the derivatization method, separations were performed in RP-HPLC conditions on a Phenomenex Synergi 4 μm MAX-RP (250 × 4.6 mm i.d.) (Phenomenex Technologies, Torrance, CA, USA) column with a mobile phase consisting of MeOH/TEA phosphate buffer. Both methods exhibited comparable performance in terms of sensitivity, linearity, precision, and accuracy, where very low % RSD and recovery values were observed; however, the limit of quantification (LOQ) achieved through derivatized glutathione analysis was five times lower than that obtained with the HILIC method.

A more recent work by R. K. Ganda et al. discusses the development and validation of a novel stability-indicating RP-HPLC-UV method for the quantification of tofacitinib citrate and two related substances (Impurity-A, Impurity-B) [48]. The method integrates HILIC technology by employing a ZIC-HILIC column as the stationary phase, which is beneficial in the retention and separation of ionizable compounds. Furthermore, the whole study involved solution stability, where the solutions were stable up to 24 h at room temperature, and forced degradation by exposing tofacitinib to acid, base, oxidation, hydrolysis, thermal and photolytic stress conditions. The data obtained resulted in a stability-indicative method as degradation products were specific and did not co-elute with any other specified peaks. Except for the aforementioned terms, the proposed technique offered a short runtime of 20 min, cost-effectiveness compared to other reported methods, robustness, reproducibility, and increased sensitivity. Thus, the method was found suitable both for batch release analysis and stability studies.

In a similar manner, S. Abu-Lafi et al. also developed and validated a stability-indicating HILIC-UV method for the determination of sulfaquinoxaline sodium in the presence of sulfaquinoxaline-related compound A in a commercially available water-soluble powder formulation [49]. The HPLC method was demonstrated to be efficient in terms of time and cost, given that the total runtime was about 10 min. The main parameters affecting the retention and separation of the analytes (e.g., peak tailing factor, resolution, etc.) were studied and optimized, while the method adequately met the accepted system suitability requirements. A forced degradation study was carried out by exposing the sulfaquinoxaline sodium standard and water-soluble powder formulation to thermal, photolytic, oxidative, and acid–base hydrolytic stress conditions. The developed HILIC method was evaluated according to the ICH/USP guidelines and proved to be accurate, very sensitive, and stability-indicating for the determination of sulfaquinoxaline sodium in its water-soluble powder in the presence of excipients, related substance A, and other degradation products. Thus, it could be adopted for quantitative quality control and routine analysis of sulfaquinoxaline sodium water-soluble powder formulations.

Another stability-indicating mixed-mode HILIC-UV assay was reported by A.R. Khatri et al. for the quantification of sodium cromoglicate in ophthalmic solutions [42]. Parameters affecting the HILIC retention mechanism, such as the column, organic constituent, ionic strength, and pH of the mobile phase, were investigated in detail. The optimal chromatographic conditions involved an Atlantis HILIC-Si column 25 cm × 4.6 mm, packed with 5 μm silica particles (Waters Corporation, Wexford, Ireland), which provided a sharp peak shape without tailing, and a mobile phase consisting of a mixture of ACN and buffer (30 mM NH_4_Ac, pH adjusted to 3.0 with acetic acid) at a ratio of 86:14, *v*/*v*. An increase in buffer strength (NH_4_^+^) resulted in greater retention of the drug. This effect is attributed to the reduction of repulsive electrostatic interactions between negatively charged silanol groups on the stationary phase and the anionic cromoglicate, due to increased ionic shielding. Conversely, increasing the pH of the mobile phase led to decreased API retention, likely due to a higher density of negatively charged silanol groups, which enhanced electrostatic repulsion with the drug. The developed methodology was validated according to ICH guidelines followed by forced degradation studies, where degradation of sodium cromoglicate was stimulated by subjecting the solutions to stress conditions. The developed HILIC method demonstrated rapidity, specificity, accuracy, and precision in the quantification of the API in ophthalmic formulations. Moreover, the authors highlighted its compatibility with ESI-MS, contributing to improved analytical sensitivity.

M. S. Ali and co-workers developed a stability-indicating method employing mixed-mode HILIC for the simultaneous determination of metformin and its related impurities, cyanoguanidine and melamine, in both bulk drug substances and finished dosage forms [44]. To minimize UV interference at the detection wavelength (218 nm), sodium dihydrogen phosphate was selected as the aqueous buffer component of the mobile phase, replacing NH_4_Ac and ammonium formate. Key chromatographic parameters—including pH, ionic strength, and the organic content of the mobile phase—were systematically optimized to achieve effective separation within a runtime of under 13 min. Method validation was conducted in accordance with ICH guidelines and was complemented by comprehensive forced degradation studies. These included thermal degradation at 105 °C for 30 days, acid and alkaline hydrolysis using 0.1 N HCl and 0.1 N NaOH, respectively, neutral hydrolysis with water, oxidative stress using 3% *w*/*v* H_2_O_2_, and photolytic degradation via exposure to UV light (254 nm) for 30 days. The method demonstrated strong analytical performance and was simple, rapid, specific, accurate, and sensitive for the quantification of metformin and its impurities.

F. Slavica and her collaborators worked on developing a fast and simple HILIC-UV method for the analysis of moxonidine and its four impurities (A, B, C and D) in pharmaceutical dosage form [46]. They employed central composite design in order to systematically examine the retention behavior of the analytes in the HILIC system and optimize the chromatographic conditions. The retention factors of moxonidine and its four impurities (*k*_M_, *k*_A_, *k*_B_, *k*_C_, and *k*_D_), as well as the resolution between impurities A and B (Rs_A/B_) and between impurities C and D (Rs_C/D_), were used as dependent variables. The critical investigated factors—percent of ACN in the mobile phase, pH of the aqueous phase, and concentration of ammonium formate in the aqueous phase—along with their interactions, were considered as independent variables. The experimental data indicated that the optimal conditions for analyte separation were a mobile phase composed of ACN and a 40 mM buffer solution (80:20, *v*/*v*) at pH 2.8. Higher volume ratios of ACN significantly prolonged the total analysis time, while lower proportions markedly reduced the resolution of the tested compounds. The selected pH value is justified by the improved ionization efficiency of the compounds in their cationic forms under acidic conditions. Compared to the official pharmacopeial method, which uses octylsilyl particles as the stationary phase and ion-pairing agents in the mobile phase, the developed method achieved adequate retention and separation of the compounds within a 12 min runtime. Additionally, the ionized compounds exhibited higher affinity for the polar stationary phase.

M.G. Weller and his research group developed a HILIC-UV-based method for the quantification of N-hydroxysuccinimide (NHS) and N-hydroxysulfosuccinimide (sulfo-NHS) [51]. NHS esters have been considered as the most important activated esters utilized in many different bioconjucation techniques. Due to their susceptibility to air moisture and water traces in solvents, the quantification of NHS would be a very helpful approach to identify reagent impurities or degradation of stored NHS esters. For the isocratic separation of NHS and sulfo-NHS, a zwitterionic silica-based HILIC column (Thermo Syncronis HILIC, (Thermo Fisher Scientific Inc., Waltham, Massachusetts (MA), USA, 150 mm × 3 mm, 3 μm) was utilized as well as a mobile phase consisting of 90% ACN and 10% of 10 mM aqueous NH_4_Ac (pH 7.5) at a flow rate of 0.4 mL/min at 30 °C. The described method was validated and applied for the analysis of NHS impurities in biotin NHS ester, Cyanine5 (fluorescent dye) NHS ester samples, and hydrolyzed biotin NHS ester samples. The authors underlined the universality of the developed approach as an important advantage, since the structurally variable ester compound is not monitored, but rather the constant degradation product NHS or sulfo-NHS, which overcomes the necessity to optimize the separation conditions and facilitates calibration considerably.

**Figure 2:**
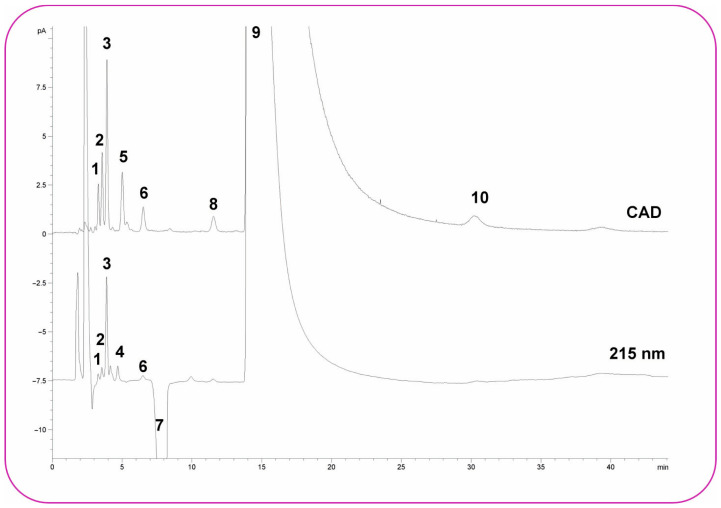
Representative chromatograms of the analysis of bicisate solution (5 mg/mL) spiked with 0.1% TFA (peak 1), l-thiazolidine-4-carboxylic acid ethyl ester (peak 4), N-methyl-cysteine (peak 2), and N,N′-ethylene-bis-l-cysteine (peak 6). (Adopted from [53] with permission.)

U. Holzgrabe et al. developed an alternative HPLC-based method for impurity profiling of N,N-ethylenebis-L-cysteine diethyl ester (bicisate) [53]. The approach employed UV detection at 218 nm followed by charged aerosol detection (CAD) using a zwitterionic HILIC stationary phase (150 mm × 4.6 mm, 5 μm particle size, 100 Å pore size) (SIELC Technologies, Prospect Heights IL, USA). The combined UV-CAD setup enabled sensitive detection of both known and unknown related substances. Isocratic separation was achieved using a mobile phase consisting of 7.5 mM TFA in ACN–water (47.5:52.5, *v*/*v*) at a flow rate of 1.0 mL/min. A representative set of chromatograms of bicisate solution spiked with the respective impurities are shown in Figure 2. Peak identities and purities were further confirmed by LC-MS/MS analysis. Method validation demonstrated satisfactory specificity, linearity, range, precision, accuracy, LOQ, and robustness. Due to the instability of bicisate in aqueous solution, sample analysis was conducted immediately after preparation. The method was successfully applied to the analysis of five commercial batches.

Later on, J. R. Denton et al. designed a HILIC-UV strategy for the trace analysis of 2-chloromalonaldehyde (2-CIMA), a potential mutagenic impurity, in active pharmaceutical ingredients [56]. An Acquity UPLC BEH Amide Column (150 × 2.1 mm, 1.7 μm) (Waters Corporation, Wexford, Ireland) was selected for further analytical method development based on the enhanced retention and selectivity observed for 2-ClMA on the BEH amide stationary phase under equivalent HILIC chromatographic conditions. Chromatographic parameters influencing the peak shape and retention time of 2-ClMA—including the concentration of NH_4_Ac in the aqueous mobile phase, column temperature, and flow rate—were systematically evaluated and optimized to prevent peak overlap with co-eluting sample components. Method attributes (LOD, LOQ, linearity) were also evaluated. Recovery studies were conducted at three impurity levels by spiking both the active pharmaceutical ingredient (API) and the first isolated API intermediate into the 2-ClMA solution. Higher recovery rates were observed for the API intermediate (97–105%) compared to the API matrix (79–107%). Overall, the HILIC-based approach mitigated several limitations typically associated with conventional RP-HPLC methods.

M. Douša developed a rapid analytical procedure for the determination of 2-aminoisobutyric acid (2-AIBA) in enzalutamide bulk drug substance based on hydrophilic interaction chromatography with fluorescence detection [45]. The method was designed to avoid a derivatization step using HILIC separation of 2-AIBA in its native form and post-column derivatization with o-phthaldialdehyde/2-mercaptoethanol. Separations were performed on a COSMOSIL HILIC column (250 × 4.6 mm, 5 μm) (Chromservis, Czech Republic). Since the influence of chromatographic conditions had been studied earlier, the optimization of the method was focused on the examination of the mobile phase composition (potassium acetate/ACN) in order to achieve the pre-set separation criteria (2-AIBA k_min_ ≥ 1.0 and 2-AIBA/3-aminoisobutyric acid resolution R_S_ ≥ 1.5). The parameters affecting the post-column derivatization reaction (pH, coil temperature, reagent concentration, and flow rate) were thoroughly investigated in order to achieve the highest detection response and reach sufficient detection sensitivity. The proposed HILIC-FLD method demonstrated good selectivity, prevented loss of the target analyte, and enabled rapid quantification of 2-AIBA in the enzalutamide bulk drug substance.

Q. B. Cass et al. reported a simple isocratic HILIC-UV method based on orthogonal separation for the simultaneous determination of iodixanol and its related impurities C, D and E in a drug substance [47]. Chromatographic separations were carried out with a Kinetex HILIC column (150 × 4.6 mm, 2.6 μm, 100 Å) (Phenomenex Technologies, Torrance, CA, USA), using ACN and aqueous FA solution (1.0 mmol/L, pH 3.2) (92:08, *v*/*v*) as the eluent at a flow rate of 0.8 mL/min and at a column temperature of 20 °C. The stationary phase was based on fused-core particle technology, which usually facilitates improved chromatography performance due to better mass transfer, in comparison to sub-2 μm fully porous particles. The column temperature was also considered an important parameter, not only for the selectivity in the HILIC column but also for the interconversion ratio between the two stereoisomeric forms of iodixanol. The temperature of 20 °C was the one that afforded the highest selectivity. The analytical method was validated for assays and related substances in line with ICH guidelines. Unlike the official USP monograph, the reported method enables the simultaneous analysis of impurities C, D, and E in a single run.

Likewise, W. Li and collaborators developed an orthogonal method using HILIC and RP-HPLC for the determination of one pharmaceutical compound and its three related impurities [39]. For the HILIC method, a YMC-pack Diol-120-NP column (250 × 4.6 mm, 5 μm) (YMC Co., Ltd., Kyoto, Japan) was utilized, while for RP-HPLC, a Waters XTerra MS C_18_ column was used (100 × 4.6 mm, 3.5 μm) (Waters Corporation, Wexford, Ireland). Regarding the HILIC method, parameters influencing retention, separation, peak shape, and elution order—such as ACN content and salt concentration—were thoroughly investigated and optimized. The elution order of the four compounds in HILIC differed significantly from that in RP-HPLC, demonstrating a high degree of practical orthogonality. In conclusion, HILIC offered distinct selectivity compared to RP-HPLC and may serve as a valuable tool for orthogonal method development.

A more recent advancement in orthogonality method development was reported by K. Yoshida et al. [62], who proposed a comprehensive strategy for impurity profiling of four synthetic cyclic pharmaceutical peptides. Their approach integrated both HILIC and RP-HPLC to maximize impurity detection and resolution. Chromatographic optimization was guided by Derringer’s desirability function, incorporating five key criteria: peptide purity, impurity resolution, peak symmetry, theoretical plate number, and retention time. For the HILIC mode, three polymeric DCpak columns (250 × 4.6 mm, 3 µm) (Daicel Corporation, Osaka, Japan) with acidic, basic, and zwitterionic stationary phases were evaluated, while RP-HPLC utilized an ACQUITY UPLC BEH C18 column (150 × 2.1 mm, 1.7 µm) (Waters Corporation, Milford, MA, USA). ACN served as the organic component in both modes, and a range of aqueous phase additives were tested, including NH_4_Ac at various pH levels, TFA, acetic acid, and ammonium hydroxide. The authors emphasized the enhanced impurity separation and improved analytical accuracy achieved by employing HILIC and RP-HPLC modes in parallel. This dual-mode methodology demonstrates strong potential for multidimensional liquid chromatography applications in pharmaceutical analysis.

B. Jancic-Stojanovic and collaborators have conducted extensive work on developing HILIC methods. In one study, they reported the analysis of olopatadine, its E-isomer impurity, and benzalkonium chloride in an eye drop formulation using HILIC [63]. Four stationary phases—cyano, amino, silica, and diol—were evaluated, with the cyano column providing the best balance of analysis time and peak shape and thus being selected for further method development. To optimize chromatographic performance, a design of experiments (DoE) approach was employed. Key factors investigated included the ACN content, pH of the aqueous phase, and NH_4_Ac concentration. A Box–Behnken experimental design was used to structure the study, with retention factors of the analytes defined as the primary responses. Multi-objective robust optimization combined with grid search techniques led to the determination of optimal chromatographic conditions, which were subsequently verified experimentally. The developed method was fully validated and demonstrated to be suitable for routine quality control. In the same year, an Analytical Quality by Design (AQbD) approach was reported for the analysis of iohexol and its impurities [50]. The relationship between critical process parameters (ACN content, the pH of the aqueous phase, and the NH_4_Ac concentration) and critical quality attributes was established using a DoE approach created in Modde 10.1 software. Monte Carlo simulation was applied to determine both model uncertainty and the variability of process parameters, supporting the development of the design space and enabling robust optimization. A year later, the same group of authors took advantage of AQbD principles to develop a HILIC method for the separation of bilastine and its degradation impurities [40]. After optimization of the separation conditions using DoE, the analytes were separated on a Luna HILIC (100 × 4.6 mm i.d., 5 μm) (Phenomenex Technologies, Torrance, CA, USA) column using a mobile phase consisting of ACN/NH_4_Ac (50 mM, pH = 5.3), 90.5:9.5, *v*/*v*. It was concluded that the ACN content in the mobile phase had a strong influence on the retention of the last-eluting analyte and therefore on the total analysis time. An increase in ACN concentration resulted in stronger retention, which is consistent with the theoretical principles of HILIC. The method was applied successfully to the analysis of Nixar^®^ tablets containing 20 mg of the API. A related study by the same research group explored the retention mechanisms of amlodipine besylate, bisoprolol fumarate, and their impurities on three different HILIC columns: amino, diol, and bare silica [59]. The authors applied theoretical models—including partitioning and adsorption models—based on thermodynamic principles to predict analyte retention behavior and underlying mechanisms, as well as the influence of mobile phase composition. The results indicated that on the amino column, retention was governed predominantly by a partitioning mechanism. In contrast, on the diol and bare silica columns, adsorption processes were primarily responsible for retention. For the amino column, a higher concentration of NH_4_Ac buffer was required to neutralize the positively charged stationary phase and suppress electrostatic repulsion with the basic analytes. Retention on the diol column involved hydrogen-bonding interactions between the analytes and the stationary phase, while on the silica column, ion-exchange interactions played a significant role. Across all three columns, increasing the ACN content in the mobile phase led to longer retention times, consistent with a reduction in the elution strength of the mobile phase. A rather similar approach has been proposed by Kuragic-Vujanovic for the separation of amlodipine basylate and its impurities [57]. The optimal chromatographic conditions were achieved using a ZORBAX NH_2_ analytical column (250 × 4.6 mm, 5 μm) (Agilent Technologies, USA) with a mobile phase composed of ACN and an aqueous solution of 50 mM NH_4_Ac (pH = 4.0) in a 90.5:9.5 (*v*/*v*) ratio. The column temperature was 30 °C, and the flow rate was set at 1.0 mL/min. As all other validation parameters were also found to be satisfactory, the proposed method is considered suitable for the determination of amlodipine besylate and its impurities in routine laboratory settings under varying conditions.

In 2019, Kasagic-Vujanovic at el. proposed a HILIC method for the separation of four impurities (Imp A, Imp B, Imp C, and Imp D) of amitriptyline API [60]. During the preliminary phase of the study, the effects of column temperature (20–40 °C), mobile phase flow rate (0.5–1.5 mL/min), and detection wavelength (190–400 nm) were investigated for all five target compounds. An increase in both temperature and flow rate resulted in reduced elution times for all analytes. This trend aligns with previously reported behavior in HILIC systems [66]. Adequate LODs were achieved in the range of 0.1–0.5 μg/mL and the recoveries were between 94.40 and 101.98%. Analogously, D. K. Ratković et al. investigated the retention mechanisms of amitriptyline and its six impurities on amide, amino, diol, and silica columns in HILIC mode [58]. They proposed dual HILIC/RP-HPLC retention mechanisms and identified transition points between HILIC and RP-HPLC behaviors on all tested columns. Partitioning and adsorption models were applied in both HILIC and RP-HPLC regions to support the evaluation of retention mechanisms. On the silica column, cation-exchange interactions were found to dominate the retention of ionized analytes, with retention increasing as pH rose. Additionally, a Box–Behnken design was employed to assess the influence of three experimental factors—ACN content, buffer pH, and buffer ionic strength—on the retention of basic compounds. This quadratic model enabled the quantification of both individual effects and two-factor interactions, particularly between ACN content and buffer ionic strength, as well as between ionic strength and pH. The study concluded that ACN content had the most significant impact on retention, followed by buffer ionic strength and buffer pH.

M. Talebi et al. developed and validated a HILIC method for the optimized separation of acetylsalicylic acid, its major impurity salicylic acid, and ascorbic acid in pharmaceutical formulations [41]. Using response surface methodology and Derringer’s desirability function, the method was optimized on a Zorbax RX-SIL silica column (250 × 4.6 mm, 5 μm) (Agilent Technologies, Wilmington, DE) with a guard column, employing an isocratic mobile phase of ACN and NH_4_Ac buffer. A Box–Behnken design was applied to model the system and identify significant parameters by simultaneously optimizing resolution and retention time. The model showed strong predictive power (R^2^ > 0.92, n = 27). Four independent variables were studied at three levels: ACN content, buffer pH, buffer concentration, and column temperature. Buffer concentration and its interaction with pH had the most significant positive effects on separation. Optimal conditions were achieved with an ACN/22 mM NH_4_Ac mobile phase at pH 4.4 (82:18, *v*/*v*) and a column temperature of 28 °C. The method revealed that retention is governed by a balance between hydrophilic partitioning and ionic interactions. Validation confirmed its suitability for analyzing the target compounds.

A zwitterionic HILIC method for quantifying L-ascorbic acid 2-phosphate magnesium (APMg) in commercial products has been reported by B. De Spiegeleer et al. [54]. The method utilized a mixed-mode HILIC–ion-exchange approach with a zwitterionic stationary phase containing embedded cation- and anion-exchange functionalities. The assay demonstrated excellent linearity (R^2^ = 0.999), precision (RSD = 0.49%), and accuracy (recovery = 100.4%). Application to five commercial APMg products revealed differing impurity profiles. Atomic absorption spectroscopy confirmed magnesium binding to the stationary phase, necessitating a strong mobile phase for effective column rinsing. Four impurities were detected: one was identified as ascorbic acid using a reference standard, while the remaining three were confirmed by MS/MS, HRMS, and NMR as ethylation products of APMg.

Y. Xu and collaborators worked on the isolation and identification of four major impurities in capreomycin sulfate [55]. The authors established and used an improved ion-pairing method to investigate the impurity profile of capreomycin sulfate substances. The developed method aimed to detect more impurities in capreomycin sulfate substances than the ion-pairing method previously reported in the literature [67]. Besides the four main components (IA, IB, IIA and IIB), four impurities (impurity A–D) with their contents much higher than the identification threshold were observed. The ion-pairing method involved the use of a Hypersil BDS C18 column as the separation column. The mobile phase consisted of ACN–phosphate buffer at pH 2.3 with 25 mM HSNa, (A) (5:95, *v*/*v*) and (B) (15:85, *v*/*v*). Furthermore, a two-dimensional (2D) LC quadrupole–time-of-flight (Q-TOF) MS method was established to realize high-resolution MS analysis of these impurities. The first-dimension LC was operated by using the improved ion-pairing method. The second-dimension LC method was performed using an Agilent ZORBAX SB C18 column. For the purpose of preparative isolation, a hydrophilic interaction chromatography (HILIC) method was established. Several HILIC columns with different stationary phases were first investigated. An Achrom Click Ion column provided the best separation performance. The HILIC method could provide good separation for the four main components of capreomycin, but the four impurities were co-eluted with each other or with the major component IB by the HILIC method. Fortunately, the degradation experiments revealed that IA and IB could yield clean impurity A and B respectively in acidic medium as well as impurity D and C respectively in alkaline medium. Therefore, IA and IB were first isolated by the preparative HILIC method, then pure IA and IB underwent acid degradation and base degradation separately, followed by re-isolation by the HILIC method to obtain pure impurity A–D, respectively. The structures of the four isolated impurities were definitely identified by Q-TOF MS and NMR analysis.

U. Holzgrabe and A. Leistner evaluated alternative approaches to the Ph. Eur. “Related substances” test using acarbose as a model API [64]. Given acarbose’s weak UV absorbance at 210 nm on aminopropyl-silyl phases, the researchers explored the use of CAD with volatile mobile phases as an alternative detection strategy. Since direct method transfer to CAD-compatible solvents failed, they screened more robust columns—including aminopropyl-silyl (UV vs. CAD), polyamine (UV/CAD), pentafluorophenyl propyl (PFP, CAD), amide HILIC (CAD), and graphite (thermal-stable)—to identify suitable alternatives. The PFP–CAD method enabled rapid total impurity quantitation, though individual peaks co-eluted; it also revealed maltose/maltotriose-related impurities. Both the graphite and amide HILIC methods were suitable for the replacement of the current Ph. Eur. method. However, for the graphite method, peak identification must be confirmed.

Likewise, R. Cirilli et al. developed an alternative HILIC method for the direct enantioseparation of oxaliplatin, addressing the limitations of the Ph. Eur. method such as low resolution and long analysis times [65]. (R,R)-oxaliplatin, the enantiopure anticancer active pharmaceutical ingredient, was separated from its (S,S)-enantiomer using a cellulose-based polysaccharide chiral stationary phase (Chiralpak IC-3, 100 × 4.6 mm I.D.) at 40 °C. The mobile phase comprised ACN–water (100:5), and separation was achieved within 8 min at a flow rate of 1 mL min^−1^, yielding a baseline resolution of 5.8. The method demonstrated high sensitivity, with LOD and LOQ for the enantiomeric impurity determined as 0.07 and 0.21 μg mL^−1^, respectively.

An AQbD-guided HILIC-UV method for the determination of imidazole (imp E) in sildenafil API and its finished products was recently reported by the research group of C. Zacharis [52]. According to the authors, the method is a viable alternative to the thin-layer chromatography (TLC) pharmacopeia method, and it was proved to be beneficial as the API and other related substances (Imp A and D) eluted almost exclusively in the void volume of the stationary phase while the polar analyte was well-resolved from other impurities at a reasonable analysis time. Ishikawa plots were utilized to visualize the high-risk method parameters, and the separation conditions were optimized using a Box–Behnken design. The method was thoroughly validated using the total-error concept approach according to “Société franc¸ aise des sciences et techniques pharmaceutiques” harmonization guidelines. Finally, the method was applied to the analysis of imidazole in various sildenafil-containing products from Bausch Health^®^ (Dublin^,^ Ireland), Lyofin® (Athens, Greece), Heremco® (Athens, Greece), Mylan Pharmaceuticals® (Dublin, Ireland), and Sandoz® (Kundl, AT, Austria) GmbH pharmaceutical companies.

In a recent study, V. D’Atri and colleagues evaluated zwitterionic HILIC stationary phases as alternatives to ion-pair RP-HPLC for oligonucleotide analysis [68]. Seven zwitterionic HILIC columns were assessed alongside amide- and polyhydroxy fructan-based HILIC columns and a C18 column. By analyzing the retention behavior of representative small-molecule pairs, each zwitterionic column exhibited a distinct radar-shaped selectivity profile, indicating unique structural discrimination capabilities. The retention of unmodified DNA and RNA samples was then studied to classify the columns based on their interaction strength. Two zwitterionic phases showed particularly strong performance, offering superior resolution for large oligonucleotides (>40-mer). The method was further applied to separate a chemically modified, drug-like ON from structurally related impurities. While the ZIC-cHILIC column displayed selectivity comparable to the IP-RPLC reference, a general decline in selectivity was noted across all columns when applied to highly complex samples with subtle structural differences. The authors emphasized the potential of zwitterionic HILIC for oligonucleotide analysis and underscored the importance of understanding column-specific retention and selectivity characteristics when selecting a stationary phase for targeted applications.

### 3.2. HPLC-CAD

The charged aerosol detector (CAD) is primarily used in liquid chromatography to detect analytes with weak or no UV absorbance. The operating principle relies on nebulizing the eluent using the Venturi effect, created by a carrier gas—usually N_2_—that flows alongside the eluent. This process converts the liquid phase into fine droplets. The droplets are then transported by the gas stream into a heated drift tube, where the solvent evaporates, leaving behind a cloud of non-volatile particles. Detection occurs by electrically charging the aerosol and measuring the charged particles with an electrical aerosol analyzer. Compared to the evaporative light-scattering detector (ELSD), the CAD has been reported to provide approximately 10 times greater sensitivity [69].

A HILIC-CAD method has been reported by Z. Long et al. for the impurity profiling of apramycin [70]. Compared to ion-pair RP-HPLC, better separation efficiency of sixteen impurities was obtained using a cysteine-bonded zwitterionic column. A higher ammonium formate concentration (100 mM) produces narrower peaks; however, a lower concentration is preferred when using mass spectrometry (MS). To remove the interference from the non-volatile SO_4_^2−^ anion, an anion-exchange solid phase extraction (SPE) method (Dionex II A cartridge) was utilized, achieving a recovery rate higher than 90%. Although the precision and the accuracy of the method were acceptable, a relatively high LOD of 8 mg/L was achieved. Compared to the official pharmacopeial method, the method is more sensitive and provides better selectivity for apramycin and related compounds.

The research group of Q. Xu, from the compendial method laboratory of USP, proposed a HILIC approach for the separation of *a*-hydroxyamine-based impurities of metoprolol [71]. These compounds (referred to as impurities M and N according to the European Pharmacopoeia (EP)) are poorly retained in RP-HPLC, lack chromophores in the UV region, and are analyzed using the official EP thin-layer chromatographic method. Various stationary phases were tested, including amide, diol, amino, polysuccinimide, etc. It was concluded that the HALO penta HILIC stationary phase provided the best chromatographic characteristics. The Halo Penta-HILIC column employs a proprietary bonding chemistry that provides a highly dense and polar bonded phase, effectively reducing ion-exchange interactions with residual silanols and enhancing both peak shape and reproducibility [72]. The method accuracy was satisfactory (94–106%) with an LOD of 80 ng/mL for both impurities. Using the proposed approach, the authors concluded that impurity N was produced under oxidation conditions, and the method could be an alternative to the pharmacopeial TLC one. Three years later, the same group of authors utilized almost the same chromatographic method as [71] for quantitative analysis of impurity N in metoprolol drug products. Forced degradation of the API was carried out to confirm the generation of the respective impurity. After validation, the method was applied to the determination of impurity N in metoprolol tartrate and metoprolol succinate extended-release products [73].

A comparative study of ion-pair RP-HPLC and HILIC in combination with CAD for the separation of underivatized amino acids was reported by U. Holzgrabe [74]. Various ion-pair reagents were examined including TFA, pentafluoropropionic acid, heptafluorobutyric acid, and nonafluoropentanoic acid in IPC and compared to acetate buffers in HILIC. Applying l-optimal design, it was revealed that much higher sensitivity (more than double) was achieved in HILIC mode than in ion-pair RP-HPLC. According to the authors, HILIC column bleeding partially counteracted the enhanced response achieved by the HILIC methodology.

More recently, a DOE-guided approach has been developed for the separation of impurities in etimicin [75]. The pH of the mobile phase and the ammonium formate concentration played a critical role in the separation of impurities. Stability studies of the etimicin solution indicated that they were stable in the time interval of 5 days. The determination coefficients of all compounds were excellent (>0.999) and the LODs were 0.15 μg/mL for all impurities. The proposed HILIC-CAD method enabled the quantification of impurities at concentrations of no less than 0.05%. The mobile phase avoids the use of ion-pairing agents such as TFA or sodium octanesulfonate, making it more compatible with both the chromatography column and the detector.

### 3.3. HPLC-MS

Coupling HILIC with MS further enhances its analytical capabilities. MS provides high sensitivity and selectivity, allowing for the accurate detection and structural elucidation of pharmaceutical compounds even at trace levels. The combination of HILIC and MS has become essential in drug development, quality control, and pharmacokinetic studies, where accurate characterization of drug substances and related compounds is critical [76].

HILIC-MS has emerged as a valuable technique for the analysis of genotoxic impurities, offering high sensitivity and selectivity for polar and trace-level compounds. An overview of HILIC-UV/FLD applications is summarized in Table 2.

Dimethyl sulfate is a commonly used alkylating agent in the organic synthesis of APIs that are highly genotoxic. Three approaches have been reported for the quantitation of this impurity in pharmaceuticals [77,78,79]. N. Grinberg et al. developed a HILIC-MS method for its determination in the starting materials used for the synthesis of metoprolol [77]. The authors proposed two analytical approaches for the determination of the analyte through derivatization with (i) dibenzazepine in 1-butyl-1-methylpyrrolidinium bis(trifluoromethylsulfonyl)imide and 1-butyl-4-methylpyridinium tetrafluoroborate ionic liquids or (ii) pyridine in ACN. It was found that enhanced reaction kinetics were observed at 120 °C and 80 °C when ionic liquids and ACN were used as the reaction medium, respectively. The dibenzazepine derivatives were analyzed using RPLC, while the pyridine-based ones were analyzed with HILIC. The determined concentration of the impurity in the analyzed samples fell within the acceptance limit of ±20%. In the same fashion, electrospray positive ionization MS/MS in combination with HILIC was reported for the determination of dimethyl sulfate in aminophenazone, caffeine, and tegafur samples [78]. A Waters Atlantis HILIC C18 column and a mobile phase consisting of 10 mM NH_4_Ac acidified methanol (with 0.1% FA) were utilized for the separation of the analyte. After method validation, excellent linearity (*r* = 0.9997) was recorded in low ppb levels with adequate relative recoveries (94.9–106.4%). More recently, single-quadrupole MS was employed after HILIC separation for the quantitation of dimethyl sulfate in pantoprazole sodium sesquihydrate samples [79]. To stabilize the analyte, the authors derivatized the analyte with triethylamine in an aprotic solvent (ACN) at 65 °C. Analogous derivatization reactions have been performed for the analysis of alkyl sulfonate dialkyl sulfate genotoxic impurities by LC-MS [80]. The analyte-triethylamine derivative was measured under single-ion monitoring at *m*/*z* 116. A high separation efficiency and a sensitivity with a limit of detection (LOD) of 0.2 ppb were achieved using ultra-high-pressure liquid chromatography (UHPLC) instrumentation. A derivatization-based HILIC-MS approach was reported by Guo et al. for the determination of dimethyl sulfate and diethyl sulfate in sulconazole nitrate samples [81]. The separation of the analytes was carried out under isocratic elution using 20 mM acidified ammonium formate and ACN at a ratio of 10/90 *v*/*v*. Multiple reaction monitoring (MRM) mode was used, and the recoveries were acceptable, being in the range of 80.3–102.3% for both analytes.

**Figure 3:**
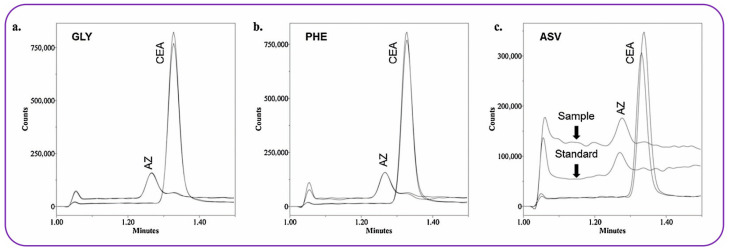
Representative SIM chromatogram from the analysis of spiked samples of (**a**) glycine, (**b**) phenylalanine, and (**c**) asunaprevir, with the impurities at 50 ppb. (Adapted from [83].)

The genotoxic impurity 2-chloro-*N*-(2-chloroethyl)ethanamine was analyzed in vortioxetine samples [82]. Various HILIC stationary phases were tested including Kinetex HILIC 2.6 μm, XBridge HILIC, Primesep B, and Obelisc R using 10 mM ammonium formate and ACN as the mobile phase. According to the authors, separation was observed only in the Primesep B column. Under optimized separation conditions, the resolution between the impurity and the API exceeded 2.5. As part of the system suitability criteria, the relative standard deviation (%RSD) of the impurity peak area was required to be ≤3.0%. In positive ionization mode, a low LOD of 0.3 ppb was achieved. A key advantage of the developed method is that the impurity is separated and quantified in its native form, eliminating the need for any time-consuming derivatization steps. A rather similar HILIC-MS approach has been developed by Shackman from the Bristol-Mayers Squibb pharmaceutical company for the quantitation of two potential genotoxic impurities (namely aziridine and 2-chloroethylamine) in three different APIs (glycine, phenylalanine, asunaprevir) [83]. Narrow analyte peaks were recorded under UHPLC conditions after a short analysis time of 1.5 min. During the calibration study, at higher levels of 2-chloro-ethylamine, a fragment ion started to appear in the aziridine selected ion monitoring (SIM) channel. Nevertheless, the chromatographic resolution was adequate (*R*_s_ = 1.4) to avoid inaccuracies (Figure 3).

**Table 2:** LC-MS applications for the determination of drug impurities.

Analyte	Analytical Column	Mobile Phase	Flow Rate (mL/min)	MS Parameters	LODs/LOQs	Recovery (%)	Reference
Dimethyl sulfate	Dihydroxypropane-bonded silica (150 × 2 mm, 3 μm)	Ammonium formate buffer (50 mM, pH = 3.2) and ACN	0.8	ESI (+ve), capillary voltage: 4 kV, SIM (*m*/*z*): 94.2	NA/0.04–0.13 μg/mL	85.4–114.5	[77]
Dimethyl sulfate	Mixed-mode silica (100 × 2.1 mm, 3 μm)	NH_4_Ac buffer (10 mM, pH = 3.2) and ACN	0.3	ESI (+ve), cone voltage: 40 V, MRM (*m*/*z*): 246.2/97.1, 230.2	0.5/1.15 ng/mL	94.9–106.4	[78]
Dimethyl sulfate, diethyl sulfate	Bare silica (50 × 2.1 mm, 3 μm)	Ammonium formate buffer (20 mM) and ACN	0.2	NA	NA	80.3–102.3 90.3–100	[81]
Dimethyl sulfate	Bare silica (100 × 2.1 mm, 1.6 μm)	Ammonium formate buffer (50 mM) and ACN	0.35	ESI (+ve), capillary voltage: 0.8 kV, SIM (*m*/*z*): 116	0.19/0.65 ng/mL	96.46–105.98	[79]
Alkyl sulfonate dialkyl sulfates	Bare silica (50 × 2.1 mm, 3 μm)	Ammonium formate buffer (50 mM) and ACN	0.3	ESI (+ve), capillary voltage: 3 kV, SIM (*m*/*z*): 88, 102, 106	NA/NA	74–137	[80]
Dimethylamine	Bare silica (150 × 3.0 mm, 2.7 μm)	Ammonium formate buffer (10 mM, pH = 4.8) and ACN	0.8	ESI (+ve), capillary voltage: 0.5 kV, SIM (*m*/*z*): 46	0.75/2.5 ng/mL	99–104.2	[84]
1,1-dimethyl-3-hydroxy-pyrrolidinium bromide	Bare silica (100 × 4.6 mm, 5 μm)	Ammonium formate buffer (10 mM) and ACN	1.2	ESI (+ve), collision energy: 27 V, MRM (*m*/*z*): 116.1/88.1	17/51 ng/mL	89.7–103.2	[85]
Degradation products of citicoline	Bare silica (50 × 4.6 mm, 3 μm)	Ammonium formate buffer (20 mM, pH = 3) and ACN	NA	ESI (+ve), capillary voltage: 4 kV, SCAN (*m*/*z*): 50–800	0.03–0.45/0.11–1.35 μg/mL	99.0–99.9	[86]
2-chloro-N-(2-chloroethyl)ethanamine	Mixed-mode (150 × 4.6 mm, 5 μm)	Ammonium formate buffer (20 mM) and ACN	0.8	ESI (+ve), capillary voltage: 0.5 V, SIM (*m*/*z*): 142	0.3/1 ng/mL	NA	[82]
Aziridine, 2-chloroethylamine	Bridged-ethyl hybrid silica (50 × 2.1 mm, 1.7 μm)	Ammonium formate buffer (100 mM) and ACN	0.5	ESI (+ve), capillary voltage: 3 kV, SIM (*m*/*z*): 44.1, 79.9	0.5 ng/mL	84–105	[83]
2,3-dichloroaniline, bis(2-chloroethyl) amine, 2-chloroethylamine	Polyhydroxyl-based silica (100 × 4.6 mm, 5 μm)	Ammonium formate buffer (100 mM, pH = 6) and ACN	0.8	ESI (+ve), capillary voltage: 3 kV, MRM (*m*/*z*): 163.1/126.3 (for 2,3-dichloroaniline), 143.1/63.1 (for bis(2-chloroethyl) amine, 80.5/63.1 (for 2-chloroethylamine)	2–30 ng/mL	96.4–98.2	[87]
Impurities in streptomycin and dihydrostreptomycin	Fused-core silica (100 × 2.1 mm, 2.7 μm)	Ammonium formate buffer (200 mM) and ACN	0.4	ESI (+ve), voltage: 4.5 kV, SCAN (*m*/*z*): 100–1000	NA/NA	NA	[88]
Amino acids, non-amino acids	Ζwitterionic silica-based HILIC (150 × 3 mm, 3 μm)	Ammonium formate buffer (100 mM) and ACN	0.5	ESI, capillary voltage: 0.3 kV (for (+)ve mode), 0.8 kV (for (*−*)ve mode), SIM (*m*/*z*): 139.0 (urocanic acid), 127.0 (4-imidazoleacetic acid), 133.0 (asparagine), 112.1 (histamine). 142.1 (histidinol), 156.1 (histidine), 175.1 (arginine) 147.1 (lysine), 132.0 (aspartic acid), 157.1 (β-Imidazolelactic acid)	1.1–18.2/2.5–42.6 ng/mL	76.1–114.3	[89]
Various impurities in parenteral solutions	Polysulfoethyl A-based HILIC (150 × 4.6 mm, 5 μm) Quinine-based HILIC (150 × 4.0 mm, 5 μm) C18 (150 × 3.0 mm, 3 μm)	Carbonate buffer (NaHCO_3_, Na_2_CO_3_, 100 mM each, pH 9.5)/ACN FA (0.1%) in water and ACN	1 mL/min 0.5 mL/min 0.3 mL/min	ESI, capillary voltage: 0.3 kV (for (+)ve mode), 0.8 kV (for (-)ve mode), MRM mode	NA/5–500 ng/mL	NA	[90]
Trimethylammonium bromide, 1,1,1-trimethylhydrazinium bromide, 3-hydroxy-1,1-dimethyl-4,5-dihydro-1H-pirazolium-1-betaine hydrate, 3-(2,2,2-trimethylhydrazinium)methylpropionate bromide, 3-(2,2,2-trimethylhydrazinium)ethylpropionate bromide and 3-(2,2,2-trimethylhydrazinium)prop-2-yl propionate bromide	Cyano-based HILIC (100 × 2.1 mm, 5 μm) Amino-based HILIC (150 × 3.2 mm, 3 μm) Silica-based HILIC (150 × 2.1 mm, 3 μm) Zwitterionic HILIC (100 × 2.1 mm, 5 μm)	Various buffers containing ammonium formate, FA, and ACN	0.2 mL/min	ESI, capillary voltage: 3 kV, MRM mode	0.00006–0.003/0.0002–0.01%	96.9–116.4	[91]
Methanesulfonic acid	Triazole-based HILIC (150 × 4.6 mm, 5 μm) Amino-based HILIC (150 × 4.6 mm, 3 μm)	Ammonium formate buffer (100 mM, pH = 3.5) and ACN	1 mL/min	ESI (-ve), voltage: 4 kV, SIM (*m*/*z*): 95 (methanesulfonic acid)	NA/NA	97.2–98.2	[92]
Semi-synthetic glycoproteins	Amide-based HILIC (150 × 2.0 mm, 3 μm)	TFA in water and ACN (0.05%)	0.3 mL/min	ESI (+ve), voltage: 4 kV, SIM (*m*/*z*): 95 (methanesulfonic acid)	NA/NA	NA	[93]
Oligonucleotide impurities and nusinersen metabolites	Amide-based HILIC (150 × 2.1 mm, 1.7 μm)	NH_4_Ac buffers at various pH values with ACN	0.4 mL/min	ESI (+ve), capillary voltage: 4 kV, SCAN (*m*/*z*): 50–1250	NA/NA	NA	[94]
Characterization of DNA and RNA oligonucleotides	Amide-based HILIC (150 × 2.1 mm, 1.7 μm)	Various NH_4_Ac and ammonium formate buffers and ACN	0.25 mL/min	ESI (-ve), capillary voltage: 3 kV, SCAN (*m*/*z*): 400–2000, MRM (*m*/*z*): 94.23 (PSO_2_^−^)	2 pmol/NA	NA	[95]
Enantiomeric impurities of cabotegravir	Cellulose-tris (4-chloro-3-methyl phenyl carbamate)-based HILIC (150 × 4.6 mm, 3 μm)	0.1% FA/ACN	1 mL/min	ESI, interface voltage: 4.5 kV (for (+)ve), −4.5 kV (for (-)ve mode), SCAN (*m*/*z*): 100–2000	0.01–0.02/0.03–0.06 μg/mL	99.2–114.9	[96]
Sequencing of oligomers	Polyvinyl alcohol-based HILIC (150 × 2.1 mm, 1.7 μm)	NH_4_Ac buffer (1 mM) and ACN	0.2 mL/min	ESI (-ve), capillary voltage: 3.5 kV, SCAN (*m*/*z*): 100–3200	NA/NA	NA	[97]
Characterization of impurities in therapeutic monoclonal antibodies	Glycoprotein amide-based HILIC (150 × 2.1 mm, 1.7 μm)	0.1% TFA in water and ACN	0.2 mL/min	ESI, capillary voltage: 4 kV, SCAN (*m*/*z*): 800–4000	NA/NA	NA	[98]

A year later, Dousa et al. developed a method for the determination of dimethylamine in metformin API and dosage forms [84]. This impurity is prone to oxidation, forming unsymmetrical dimethylhydrazine, which can further oxidize, resulting in the formation of carcinogenic *N*-nitrosodimethylamine [99]. Due to the polarity of dimethylamine, Cortecs HILIC stationary phase was utilized to enhance its retention. The API tablets were powdered and extracted using a water/ACN mixture prior to LC-MS analysis. The method was relatively fast, with a runtime of only 5 min per sample, while its linearity was increased up to 250 ng/mL. The dimethylamine concentration in the analyzed samples varied between 16 and 230 μg/g. A rather similar work has been published by S. Mullangi et al. for the quantitation of three potential genotoxic impurities in aripiprazole API [87]. The analytes were separated using gradient elution on an ACE HILIC stationary phase. For the detection of the impurities, one parent and one daughter ion were selected to ensure the sensitivity and selectivity of the determination. The precision of the method was lower than 10% (as %RSD), while no detectable impurities were recorded in the analyzed API commercial products.

A relatively fast determination of 1,1-dimethyl-3-hydroxy-pyrrolidium bromide in glycopyrrolate oral solution was achieved [85]. The authors unsuccessfully attempted to separate the impurity from the glycopyrrolate using a C18 column, and they finally adopted a HILIC approach with a short gradient program of 5 min. Forced degradation studies of the API revealed that approximately 26% of the impurity was formed under alkaline conditions (5N NaOH at 60 °C for 2 h). The method had excellent linearity in the range of 50–2000 ng/mL, while its precision (intra-day and inter-day) was less than 2.5% in all cases.

An interesting HILIC–quadrupole ion trap/time-of-flight (QIT/TOF-MS) method was reported for the detection of impurities in streptomycin and dihydrostreptomycin [88]. Compared to the pharmacopeial ion-exchange HPLC-based method, the proposed approach provided both separation and structural elucidation of the impurities. The authors concluded that the small particle size of the stationary phase inherently offered the benefit of producing very sharp peaks, but the broader peaks indicated that cation exchange was the primary retention mechanism, occurring between the deprotonated silanol groups on the column and the amino groups of the analytes. Using similar instrumentation, the research group of S. Guermouche characterized the forced degradation products of citicoline [87]. A bare silica HILIC column was utilized for the separation of impurities using a mixture of ACN and formate buffer at pH 3. Three impurities were generated under acidic, alkaline, and oxidative stress conditions, respectively, and were identified using both MS/MS and NMR techniques. Similarly, K. Maekawa et al. determined the purity of the gentamicin reference standard of Japanese Pharmacopoeia using HILIC-MS/MS [100]. The authors took advantage of MS/MS instrumentation to selectively determine the content of the gentamicin C component. The potency of the API reference standard was also determined using ^1^H qNMR and microbiological assays. A potential limitation of the HILIC method was the fact that the gentamicin C_2_ used in the assay also contains C_2a_ and C_2b_, which cannot be separated from C_2_.

In 2022, a HILIC-MS method was reported for the determination of amino acid and non-amino acid impurities in histidine raw material [89]. During histidine synthesis, 12 potential impurities including amino acids (arginine, lysine, asparagine, aspartic acid, alanine, and glycine) and their non-amino acid impurities (histamine, histidinol, 4-imidazoleacrylic acid, 4-imidazoleacetic acid, *β*-imidazolelactic acid, and urea) can be formed. It was found that increasing the buffer pH resulted in a longer retention time for aspartic acid, while it caused shorter retention times for histidine, histamine, and histidinol. Small injection volumes (1–2 μL) of the water-rich samples were selected to avoid peak broadening and tailing. Low LODs were reported in the range of 0.8–18.2 ng/mL. The method could be incorporated into the USP histidine monograph to replace the old-fashioned titration assay and the TLC method for related compounds. A rather similar approach was reported for the impurity profiling of parenteral infusion solutions for amino acid supplementation containing L-alanyl-l-glutamine [90]. Three stability-indicating LC-MS/MS methods were developed to quantitatively profile impurities. Two different HILIC analytical columns were utilized (Chiralpak QN-AX, Polysulfoethyl A) and one RP (Gemini). The main objective was to resolve isobaric compounds—including stereoisomers, constitutional isomers, and retro-peptides—and to quantify impurities detected in stressed nutritional infusion solutions. The methods were calibrated using standard addition within the samples and validated in compliance with ICH guidelines. Analysis of stressed samples showed that, in addition to deamidation of alanine-glutamine to alanine-glutamic acid and peptide hydrolysis, cyclization (diketopiperazine formation) and other condensation reactions also occur—primarily involving components present at higher concentrations.

Hmelnickis et al. developed a HILIC -MS method for the simultaneous separation of mildronate and six related impurities in API [91]. The influence of key chromatographic parameters—stationary phase, ACN content, and ammonium formate buffer pH—on retention and selectivity was systematically evaluated. Six columns were tested, including three silica-based, and one each of amino, cyano, and zwitterionic sulfobetaine types. Among these, the cyano column showed no structural selectivity, while one silica column and the zwitterionic sulfobetaine column offered balanced retention and acceptable peak shapes. Ultimately, the latter stationary phase demonstrated superior selectivity and was chosen for method validation. The validated method was successfully applied to both technical and commercial batches, confirming its suitability for routine purity analysis of mildronate API.

Z. Huang et al. developed a simple, sensitive, and robust HILIC–(ESI)-MS method for quantifying methanesulfonic acid (MSA) at low ppm levels [92]. The method was designed to verify MSA removal during pharmaceutical synthesis and to explore HILIC retention mechanisms for organic acids on positively charged stationary phases, such as amino and triazole. Several sulfonic acids—including ESA, PSA, BSA, PTSA, HSA, HPSA, and OSA—were evaluated to investigate the separation behavior. Chromatographic separation was performed using a Cosmosil HILIC column and a Unison UK-Amino column with a mobile phase of 100 mM ammonium formate–ACN (25:75, *v*/*v*) at pH 3.5. The study demonstrated that both surface adsorption and ion exchange contribute to retention under typical HILIC conditions (aqueous content 5–20%), with ion exchange becoming more prominent at higher water contents. Importantly, ESI-MS sensitivity for sulfonic acids increased markedly with higher ACN fractions (up to 95%), emphasizing the advantages of HILIC for ionizable analytes.

C. Temporini and collaborators investigated the use of HILIC coupled with high-resolution TOF-MS for characterizing intact neo-glycoproteins formed by chemically conjugating synthetic saccharides to lysine residues of selected recombinant proteins [93]. The study evaluated the separation performance of three amide-based HILIC columns using water–ACN gradients with various volatile additives. The addition of 0.05% (*v*/*v*) TFA to the mobile phase was found essential for achieving optimal glycoform resolution and minimizing ion suppression. Gradient conditions were individually optimized for each protein–column combination. The HILIC phases were tested on semi-synthetic derivatives of ribonuclease A, as well as on TB10.4 and Ag85B glycoconjugates. Results showed that HILIC selectivity was primarily influenced by the size and number of conjugated glycans, enabling effective glycoform separation. Moreover, coupling HILIC to HRMS facilitated the identification of product-related impurities. Overall, the study demonstrated that amide HILIC combined with MS is a powerful approach for the detailed characterization of intact neo-glycoproteins, allowing the determination of glycoform number, identity, and relative abundance in complex semi-synthetic products.

A cellulose-tris (4-chloro-3-methyl phenyl carbamate) chiral stationary phase was used for the enantioseparation of impurities’ isomers of cabotegravir [96]. Symmetric peaks were obtained for *R*, *R* and *S*, *S* isomers at ambient temperature using aqueous formic acid (FA) and ACN as the mobile phase. The method was validated according to ICH guidelines, and it was found to be robust by changing the mobile phase composition, the flow rate, and the column temperature. The recoveries of the isomers in three bulk drug batches were acceptable, being in the range of 99.6–100.9%. According to the authors, the method can be readily adapted for preparative-scale purifications using the chiral stationary phase, and it is suitable for routine analysis of pharmaceutical quality.

**Figure 4:**
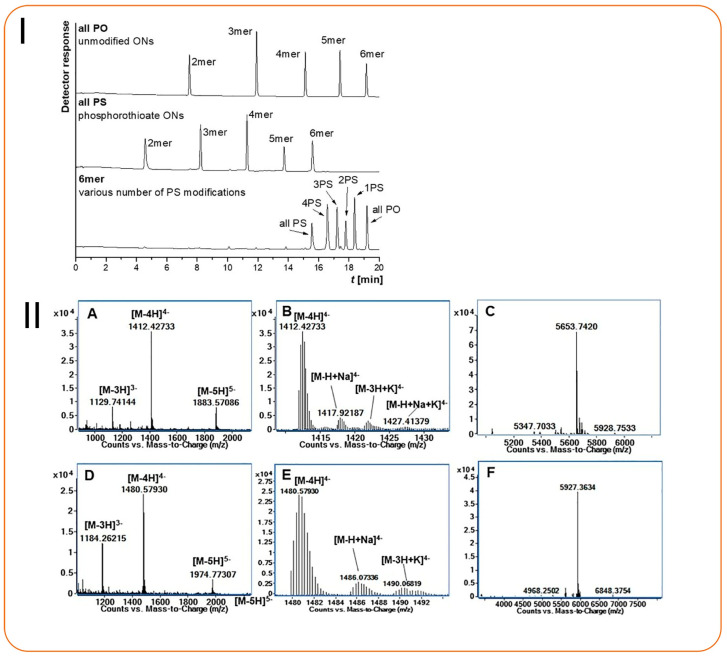
(**I**) Representative separations of oligos based on their length and number of thiophosphate substitutions; (**II**) (**A**,**D**) full scan spectra, (**B**,**E**) adducts, and (**C**,**F**) deconvoluted masses of oligo 1 and oligo 2 (reprinted from [94] with permission).

The significance and application of therapeutic oligonucleotides in gene and antisense therapies have seen substantial growth in recent years. These drugs are employed in the treatment of conditions such as spinal muscular atrophy, cytomegalovirus retinitis in AIDS patients, etc. [101]. The role of HILIC in the analysis of oligonucleotides has been comprehensively discussed elsewhere [102]. An interesting research work has been published by Z. Vosahlova et al. on the separation and characterization of antisense oligonucleotide impurities in nusinersen [94]. A quadrupole-TOF/MS (QTOF) in negative ionization mode was used for the sequence and structure elucidation of the impurities. One part of this work is devoted to the investigation of the HILIC separation conditions of 21-mer and shorter impurities as representative therapeutic oligos of the first generation. The pH of the mobile phase had a great influence on the separation: its maximum value was achieved at pH 8. More narrow peaks were also recorded at higher ammonium formate concentrations (i.e., 25 mM). A representative separation under optimum experimental conditions is shown in Figure 4I. Mongo Oligo mass calculator software (version 2.06) was utilized for sequence assignment (Figure 4II). Regarding the characterization of DNA and RNA oligos, generic HILIC-MS/MS has been proposed [95]. An amide BEH column was used, relying on hydrogen-bonding interactions with the analytes for separation. HRMS was employed to determine the deconvoluted masses of oligos and siRNA standards, along with their associated impurities. Unbiased sequence anIlysis was performed, and the higher-energy C-trap dissociation parameters were optimized to enhance the sequence coverage of both DNA and RNA oligos. Superior sensitivity was achieved by detecting as little as 13 ng (2 pmol) of the oligonucleotide. The sequence of phosphorodiamidate morpholino oligomers has been analyzed by the research group of Sarenta Therapeutics Inc. [97]. To achieve satisfactory MS sensitivity, the authors utilized a diol-based HILIC analytical column using 1 mM NH_4_Ac and ACN as mobile phases. In terms of MS parameters, the gas temperature, the nozzle voltage, and the fragmentor voltage were set to 275 °C, 2 kV, and 275 V, respectively. In positive ion mode, the phosphorodiamidate morpholino oligo 1 exhibited multiple charge states ranging from +4 to +7 with the most intense charge state of +5, which was selected as the precursor ion for collision-induced dissociation fragmentation.

The low-molecular-weight impurities in therapeutic monoclonal antibodies attracted the interest of S. Wang et al. [98]. A recombinant IgG1 monoclonal antibody drug product was used as a model molecule in this work. Following treatment with PNGase F to remove N-linked glycans from each heavy chain, the deglycosylated antibody sample was separated using a HILIC column and analyzed by both UV (280, 215 nm) and MS. A potential drawback of the HILIC method is that the resolution was inferior to the SDS-PAGE method, especially in the separation of the low-molecular-weight impurity.

### 3.4. Two-Dimensional LC

Multidimensional liquid chromatography is increasingly being adopted for situations where traditional one-dimensional liquid chromatography does not provide adequate analytical resolution. Thanks to its wide range of separation modes (such as reversed phase, size exclusion, ion exchange, and hydrophilic interaction) and the fact that nearly any sample can be dissolved in a suitable liquid medium (whether aqueous or organic), this technique is extremely versatile and widely used in both academic research and industrial applications [103]. A useful tutorial on ^2^D-LC has been published by Stoll et al. [104].

**Figure 5:**
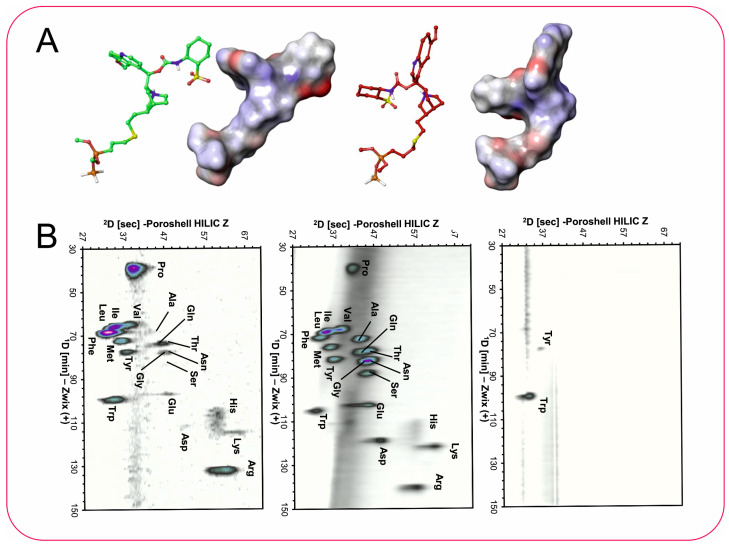
(**A**) Minimal energy conformations of the ZWIX(+) and ZWIX(−) chromatographic ligands. The balls and sticks are colored with green and red carbon atoms for ZWIX(+) and ZWIX(−), respectively. (**B**) ^2^D separation of underivatized proteinogenic amino acids using ZWIX(+) in polar organic mode in ^1^D and Poroshell 120 HILIC-Z under HILIC conditions and complementary detection in ^2^D for comprehensive sample information. (Reprinted from [106] with permission.)

An interesting method using ion-pair RP (IPRP) and anion-exchange chromatography (AEX) coupled to HILIC/MS was developed for the characterization of antisense oligonucleotide impurities [105]. The authors separately optimized the ^1^D IPRP and AEX conditions using a one-factor-at-a-time approach. It was found that the ion-pair reagent was a key parameter. The combination of hexafluoro-2-propanol and triethylamine with concentrations of 400 and 16.3 mM, respectively, provided the best separation efficiency. Looking at AEX conditions, adding 20% ACN to the mobile phase minimized secondary hydrophobic interactions between the exposed bases of the single-stranded antisense oligonucleotide and the AEX stationary phase. By carefully choosing the loop volume and the dimensions of the ^1^D and ^2^D columns, the AEX and IPRP modes were successfully coupled with HILIC, facilitating highly sensitive impurity detection using MS. In the same year, the research group of Lammerhofer studied the utilization of two Chiralpak columns (ZWIX(+) and (ZWIX(−))—based on zwitterionic quinine and quinidine carbamate selectors—in an orthogonal scheme for the impurity profiling of amino acids [106]. The authors concluded that these chiral columns function as mixed-mode chromatography phases, characterized by a lipophilic–hydrophilic surface and overall negative charge due to the sulfonic acid groups. Molecular modeling and conformational analysis were carried out to determine the minimal energy conformations, as shown in Figure 5A. The proposed ^2^D-LC method integrated with complementary detectors (DAD, CAD, and HR-MS/MS) has been successfully applied for impurity profiling of amino acids and proteinogenic amino acids (Figure 5B).

Three years later, a significant work on this topic was published in the *Analytical Chemistry* journal [107]. This work focused on a straightforward instrumental strategy involving the use of an in-line mixer to enable complex ^2^D-LC workflows, including the coupling of gel permeation chromatography (GPC) with RPLC for the analysis of hydrophobic oligomers, and the integration of IPRP with HILIC for the analysis of polar antisense oligonucleotides. More recently, a heart-cutting method utilizing IPRP-HILIC coupled with QTOF/MS, integrated with a make-up flow module, was established to analyze polar impurities in calcium gluconate injection [108]. In the first dimension, the IPRP-LC method was employed to selectively separate polar analytes using a fully aqueous mobile phase containing phosphate buffer and an ion-pairing agent. Selected peaks of interest were isolated using sample loops and transferred to the second-dimension HILIC column, which utilized an organic-rich mobile phase. To address the incompatibility between the water-based mobile phase from ^1^D and the organic-rich conditions of ^2^D, the authors incorporated a make-up flow module to dilute the water-rich fractions with ACN before they entered the sample loops. Five impurities were identified following acidic and thermal forced degradation of the API.

## 4. Conclusions

Over the past two decades, HILIC has become a vital tool for analyzing polar and hydrophilic impurities in pharmaceutical substances. Its unique retention mechanism, MS compatibility, and ability to resolve structurally similar analytes make it highly suitable for impurity profiling in both small- and large-molecule drug products. While this review highlighted the breadth of HILIC applications (including method development strategies, advances in stationary phases, and detection approaches), it is important to recognize certain limitations. Method development often requires extensive optimization due to the interplay of multiple retention mechanisms, peak shape issues with strong ionic analytes, and the high sensitivity of HILIC to mobile phase composition. Furthermore, the lack of universally applicable stationary phases restricts broad applicability across impurity classes. Looking ahead, more robust and versatile stationary phases, coupled with advances in computational modeling, machine learning, and integration with high-resolution MS and multidimensional LC, are expected to enhance predictability, robustness, and the scope of HILIC in pharmaceutical impurity profiling.

## Data Availability

No new data were created or analyzed in this study. Data sharing is not applicable to this article.

## Abbreviations

The following abbreviations are used in this manuscript:

2-AIBA	2-aminoisobutyric acid
2-CIMA	2-chloromalonaldehyde
ACN	Acetonitrile
AEX	Anion-exchange chromatography
API	Active pharmaceutical ingredients
AQbD	Analytical quality by design
CAD	Charged aerosol detector
DAD	Diode array detector
DoE	Design of experiments
ESI	Electrospray
ELSD	Evaporative light-scattering detector
EP	European Pharmacopoeia
FA	Formic acid
FLD	Fluorescence detector
GPC	Gel permeation chromatography
HILIC	Hydrophilic interaction chromatography
HPLC	High-performance liquid chromatography
ICH	International Council for Harmonization
IPRP	Ion-pair reversed phase
LOD	Limit of detection
LOQ	Limit of quantitation
MS	Mass spectrometry
NA	Not applicable
NH_4_Ac	Ammonium acetate
NHS	N-hydroxysuccinimide
QTOF	Quadrupole time-of-flight
RP	Reversed phase
RP-HPLC	Reversed-phase high-performance liquid chromatography
SIM	Selected ion monitoring
SPE	Solid phase extraction
sulfo-NHS	N-hydroxysulfosuccinimide
TFA	Trifluoroacetic acid
TLC	Thin-layer chromatography
USP	United States Pharmacopeia
